# Aegean wall lizards switch foraging modes, diet, and morphology in a human‐built environment

**DOI:** 10.1002/ece3.2501

**Published:** 2016-09-27

**Authors:** Colin M. Donihue

**Affiliations:** ^1^ School of Forestry and Environmental Studies Yale University New Haven CT USA; ^2^ Department of Organismic and Evolutionary Biology Harvard University Cambridge MA USA

**Keywords:** foraging syndrome, functional trait, Greece, human land use, hunting mode, local adaptation, *Podarcis*

## Abstract

Foraging mode is a functional trait with cascading impacts on ecological communities. The foraging syndrome hypothesis posits a suite of concurrent traits that vary with foraging mode; however, comparative studies testing this hypothesis are typically interspecific. While foraging modes are often considered typological for a species when predicting foraging‐related traits or mode‐specific cascading impacts, intraspecific mode switching has been documented in some lizards. Mode‐switching lizards provide an opportunity to test foraging syndromes and explore how intraspecific variability in foraging mode might affect local ecological communities.Because lizard natural history is intimately tied to habitat use and structure, I tested for mode switching between populations of the Aegean wall lizard, *Podarcis erhardii*, inhabiting undisturbed habitat and human‐built rock walls on the Greek island of Naxos. I observed foraging behavior among 10 populations and tested lizard morphological and performance predictions at each site. Furthermore, I investigated the diet of lizards at each site relative to the available invertebrate community.I found that lizards living on rock walls were significantly more sedentary—sit and wait—than lizards at nonwall sites. I also found that head width increased in females and the ratio of hindlimbs to forelimbs in both sexes increased as predicted. Diet also changed, with nonwall lizards consuming a higher proportion of sedentary prey. Lizard bite force also varied significantly between sites; however, the pattern observed was opposite to that predicted, suggesting that bite force in these lizards may more closely relate to intraspecific competition than to diet.This study demonstrates microgeographic variability in lizard foraging mode as a result of human land use. In addition, these results demonstrate that foraging mode syndromes can shift intraspecifically with potential cascading effects on local ecological communities.

## 1 Introduction

Foraging mode is a functional trait affecting species' impacts on ecological communities (Miller, Ament, & Schmitz, 2014; Post & Palkovacs, 2009; Schmitz, 2008; Schoener, 2011). Classically, predators were grouped according to two foraging modes: active foraging predators that course widely in search of prey, and sit‐and‐wait predators that remain relatively sedentary until ambushing their prey (Huey & Pianka, 1981; MacArthur & Pianka, 1966; McLaughlin, 1989; Pianka, 1966; Schoener, 1971). Scientists have since recognized that animal foraging modes fall along a continuum between these two extremes (Butler, 2005; Miles, Losos, & Irschick, 2007; Perry, 1999); however, species are still classified as either relatively sit and wait or active foraging when testing predictions about foraging mode‐associated morphological, performance, and behavioral traits (Miles et al., 2007; Vanhooydonck, Herrel, & Van Damme, 2007) and making predictions about their functional roles in ecological communities (Miller et al., 2014; Schmitz 2010).

Generalizing foraging modes in this way is advantageous for describing and predicting ecological community dynamics (Schmitz 2010). For example, sit‐and‐wait predators almost exclusively encounter and consume highly mobile prey, whereas active foraging predators tend to consume a higher proportion of sedentary, cryptic prey (Huey & Pianka, 1981; Vanhooydonck et al., 2007) and may supplement their diet with plant material (Herrel, 2007).

Comparative studies have also shown that a suite, or syndrome, of behavioral, morphological, physiological, and performance traits go hand‐in‐hand with foraging mode. Thus, generalizations can be made about a predator species' physiognomy and life history following identification of a predators' foraging mode, and conversely, measurement of these characteristics can enable predictions of foraging type (McLaughlin, 1989). For instance, sit‐and‐wait lizards often have wider mouths to facilitate rapid prey handling (McBrayer & Corbin, 2007). These wider heads often correspond to proportionally stronger bite forces, facilitating ingestion (Herrel, Vanhooydonck, & Van Damme, 2004; Vanhooydonck et al., 2007). Furthermore, sit‐and‐wait lizard predators tend to have longer hindlimbs relative to their forelimbs (hindlimb‐to‐forelimb ratio) that enable quick accelerations to capture passing prey (Huey & Pianka, 1981; Miles et al., 2007; Thompson & Withers, 1997). Active foraging lizard species tend to have relatively elongate heads, which increases the velocity of movement of the jaws (McBrayer & Corbin, 2007). They also tend to have a more equal hindlimb‐to‐forelimb ratio facilitating endurance runs and maneuverability on the chase (Garland & Losos, 1994; Huey & Bennett, 1986; Huey & Pianka, 1981).

Foraging mode is often considered a fixed species trait (Perry, 1999; Verwaijen & Van Damme, 2007) because the hypothesized syndrome of related traits should strongly constrain foraging mode switching (McLaughlin, 1989). This assumption lies at the heart of studies inferring hunting mode from foraging trait syndromes, and using foraging mode to predict ecological community dynamics. However, the syndrome hypothesis derives from comparative interspecific studies that are confounded by species' phylogenetic relatedness (McLaughlin, 1989; Miles et al., 2007). In other words, traits could be shared among species because they are closely related, not because they share a foraging mode. An alternative test of foraging syndromes would compare populations of the same species in different ecological contexts with consistently different behavior.

Intraspecific foraging mode switching has been documented in several animal clades (reviewed in Helfman, 1990). Among lizards, ecological context such as presence or absence of predation risk, types of prey resources available, or intensive grazing by domestic animals may cause shifts in foraging mode (Greeff & Whiting, 2000; Hawlena & Pérez‐Mellado, 2009; Wasiolka, Blaum, Jeltsch, & Henschel, 2009). For example, when five populations of the active foraging fringed lizard *Acanthodactylus beershebensis* experienced experimentally elevated predation risk from kestrels, they became relatively sit and wait, changing their diet to smaller, more active insects (Hawlena & Pérez‐Mellado, 2009). Populations of the typically sit‐and‐wait flat lizard, *Platysaurus broadleyi* living near fig trees adopt a more active foraging mode during fruiting seasons to eat dropped fruit (Greeff & Whiting, 2000; Whiting, 2007). Intensive cattle grazing caused the sit‐and‐wait spotted sand lizard (*Pedioplanis l. lineoocellata*) to become more active foragers (Wasiolka et al., 2009). While these cases have demonstrated a capacity for intraspecific foraging mode shifting to align with ecological context, it remains uncertain whether or not corresponding trait syndromes shift as well. This study explores how ecological context is related to population‐level variability in foraging mode and associated traits in the Aegean wall lizard (*Podarcis erhardii*) to test the foraging syndrome hypothesis and infer a potential evolutionary ecological basis for foraging mode changes.

Classic evolutionary theory holds that intraspecific population‐level differences in foraging mode related traits would be expected to occur only over distances that exceed the dispersal range of an animal (Lenormand, 2002). This stems from the assumption that gene flow will counteract local adaptation if genes are entering a local population from a source population experiencing different selective pressures. However, newer evidence suggests that microgeographic local adaptation of behavior, morphology, and performance may be more prevalent than previously appreciated (Richardson, Urban, Bolnick, & Skelly, 2014). If individuals distribute themselves across a landscape nonrandomly so as to match their phenotypes to local habitats that confer higher fitness, this process might enhance local adaptation (Richardson et al., 2014). The ability to match to local context may also be abetted by phenotypic plasticity in traits, as has been demonstrated in lizard and fish species (Eklöv & Svanbäk, 2006; Losos, 2009; Losos et al., 2000). Such plasticity could also be an important antecedent to rapid evolutionary change and local adaptation (Schoener, 2011).

Habitat structure often critically affects lizard fitness, and directly influences lizard behavior, morphology, and performance (Losos, 2009; Revell, Johnson, Schulte, Kolbe, & Losos, 2007; Vanhooydonck et al., 2007). As such, it follows that if habitat changes can cause foraging mode shifts (Wasiolka et al., 2009), these changes may accordingly affect the syndrome of behavioral, morphological, and performance traits that correspond to foraging mode.

I tested the hypothesis that changes in habitat structure could shift lizard foraging mode in the Greek archipelago where there is a wide variety of natural and human‐altered habitats. As part of agricultural practices, humans have built numerous stone walls that create novel habitat structure for lizards. Traditional dry (*i.e.,* lacking cement) stone walls are pervasive around olive groves and goat pastures and have been built with millennia‐old techniques (Grove & Rackham, 2003, Hughes, 2005). Agricultural habitats where stone walls are common differ considerably from the native phrygana vegetation characterizing undisturbed areas. Phrygana is a lowland habitat characteristically found through much of the Mediterranean basin dominated by short evergreen or summer‐deciduous spinose scrub, interspersed with many species of aromatic forbs. In Greece, both habitats can be densely populated by the Aegean wall lizard, *P. erhardii*.

I investigated the foraging mode and related syndrome of morphological and performance behavior traits among *P. erhardii* populations residing in wall habitats and in nonwall Mediterranean scrub. Based upon previous personal observations, I hypothesized that lizards on walls adopt a relatively sit‐and‐wait foraging mode. I then tested whether wall and nonwall populations also differed in morphology (sit‐and‐wait lizards should have a larger hindlimb‐to‐forelimb ratio and wider heads relative to nonwall active foraging lizards), bite force (sit‐and‐wait lizards have a stronger bite force), and diet (sit‐and‐wait lizards eat a higher proportion of active insect taxa).

## 2 Materials and Methods

*Podarcis erhardii* is a medium‐sized (snout‐to‐vent length 49–78 mm) lizard, widely distributed although Greece (Valakos et al., 2008). As a species, it is a habitat generalist and can be found in most habitat types throughout the region (Roca, Foufopoulos, Valakos, & Pafilis, 2009; Valakos et al., 2008). In human‐populated areas, it is often found on rock walls and terraces. It is a generalist predator of arthropods and is known to also eat snails (Adamopoulou, Valakos, & Pafilis, 1999). While frugivory has been observed for this species (Brock, Donihue, & Pafilis, 2014), its diet is largely devoid of plant material (Adamopoulou et al., 1999; Valakos, 1986).

**Figure 1:**
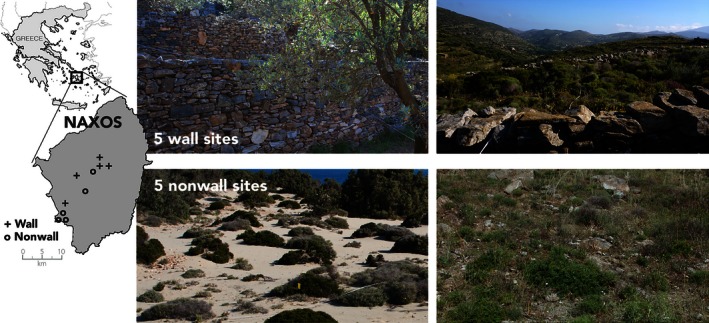
The island of Naxos in the Greek Cyclade Island cluster (inset map) and representative pictures of the habitats from the five wall and nonwall sites used for study. The approximate location of all of the wall (plus) and nonwall (circle) study sites are marked on the inset map. Exact locations can be found in Appendix 1

I conducted this study on Naxos, the largest island in the Greek Cyclade Island cluster (Figure 1). There are a wide variety of habitat types within its 440 square kilometer area, and there is a long history of human land use for agriculture (Grove & Rackham, 2003; Hughes, 2005). In uncultivated areas, Mediterranean phrygana/maquis vegetation is characteristic, composed of evergreen or summer‐deciduous, dwarf, spinose scrub, and many species of aromatic forbs. The most visible effects of human land use are habitats containing built dry‐stone (*i.e.,* lacking cement) rock walls and terraces. Terraces have been used to increase arable land on slope faces for millennia (Grove & Rackham, 2003), although most terraces in modern use were built in the nineteenth century (Hughes, 2005).

For this study, I surveyed ten populations of *P. erhardii* on Naxos from a wide variety of habitats on the island (Figure 1). Five sites were selected for their lack of human‐built rock walls. Three of these sites were located in the federally protected area Alyko (Figure 1), which is dominated by *Juniperus oxycedrus* shrubs on a sandy substrate and is devoid of built stone structures. Two additional sites were situated on rocky terrain, lacking walls, but composed of a loose jumble of stone, soil, and interspersed shrubs and forbs. To contrast, five sites were selected with built stone walls and terraces; freestanding walls averaged 1 m in height, while terraces stood between 1.5 and 2 m tall (Figure 1). These sites were situated in olive groves or phrygana. All sites were within 15 km of each other and based upon the contiguous presence of *P. erhardii* between sites and the lack of obvious barriers to gene flow on this island; there should be no spatial autocorrelation between sites.

During May and June of 2014, I created a 50 m by 50 m square plot at each site. A three‐person team then surveyed the plot for three hours during the morning high‐activity period between 0900 and 1200, noosing and capturing as many *P. erhardii* adults as possible within the plot. A total of 76 females and 94 males were caught across the wall sites, and 73 females and 81 males were caught at nonwall sites. Immediately after capture, I flushed the stomachs of each lizard using a ball‐tipped syringe until the contents of the lizard's stomach were regurgitated into a small‐mesh strainer, following standard protocols (Donihue, Brock, Foufopoulos, & Herrel, 2015; Herrel, Joachim, Vanhooydonck, & Irschick, 2006; Herrel et al., 2008). These contents were preserved in ethanol and identified at a later date. All lizards were then transported to my lodgings in the Chora of Naxos overnight for further measurements, after which the lizards were returned to their site of origin.

At each of the ten plots, I set eight sticky insect traps on a 30‐cm wire stake. At the base of the stake, I buried a pitfall trap (opening eight cm in diameter), containing two cm of antifreeze. All traps were deployed for 48 hr, at which point they were collected and the contents of each trap were later identified to order.

At each of the ten sites I also recorded videos of in situ behavior of lizards. All videos were recorded (using a handheld Sony HDRPJ260V) from at least 2 m distance during the highest‐activity morning hours between 09:00 and 12:00. All recordings at a given site were taken on a single day, and sites were only visited on hot sunny days for consistent weather conditions. To avoid duplicate recordings of the same lizard after each video recording the focal lizard was either caught for further analysis or, if the lizard could not be caught, an approximately 5 m area around the lizard was never revisited during the remainder of the recording session. I analyzed all videos by calculating the percent time each lizard spent active or sedentary (following Verwaijen & Van Damme, 2007). Furthermore, two additional scientists analyzed each video independently, and our analyses were compared to test for viewer bias. When there was discrepancy more than 1 standard deviation in estimated time active or sedentary I reanalyzed the video (2 of 94 videos). For analysis, I omitted all videos containing less than 60 s of continuous data, or where it was clear that the lizard was reacting to the presence of the recorder. Due to this constraint, I used a total of 74 videos from 7 sites (4 with walls and 3 without walls) averaging 115 s in length for statistical analysis. The minimum number of videos from a site was 7 (a nonwall site), and the median number of videos across all sites was 11.

In laboratory, I measured several morphological traits on each lizard: body size (snout‐to‐vent length—SVL); the length (snout tip to back of parietal scale), width (at widest point, including soft tissue), and height (at back of parietal scale) of the head; and the total length of both the right arm and leg (each limb segment was measured and summed). All length measures were taken using digital calipers (Frankford Arsenal Electronic Dial Calipers).

I measured bite performance of the lizards using a purpose‐built bite force meter. The meter relies on two parallel metal bite plates that pivot over a microcaliper fulcrum and induce a current in a Kistler force transducer (type 9203, Kistler Inc., Switzerland), proportional to the force of the bite (See Herrel, Spithoven, Van Damme, & De Vree, 1999 for full description). The lizards bit the plates in three consecutive trials, and the strongest bite was used for all analyses as maximum bite capacity. The distance between bite plates can vary, and greater plate distances provide a mechanical advantage for larger animals due to their relatively lower gape angle (Donihue et al., 2015; Durmont & Herrel, 2003; Herrel, Aerts, & De Vree, 1998). Thus, for each individual, I also recorded bite plate distance to be used as a covariate in all bite force analyses.

Lizard stomach contents were analyzed, and each invertebrate prey item was identified to order. These orders were assigned activity level indices (following Vanhooydonck et al., 2007 based upon observed escape behaviors. See Appendix 2), and the proportion of active and sedentary diet items was calculated for each lizard. The same activity indices were applied to the insects caught and identified in the pitfall and sticky traps at each site. Based upon the number of active and sedentary taxa in each lizard stomach, relative to the number of active or sedentary insects trapped at each site, I calculated a selection index for each lizard for active or sedentary prey. This index was calculated, following Loehle and Rittenhouse (1982), for each lizard as number of active or sedentary prey consumed divided by the number of active or sedentary prey available in the habitat (as measured by the traps). Finally, as lizard bite force is in other systems strongly related to prey hardness (Herrel & De Vree, 2009; Herrel et al., 2004, 2008), I also assigned a hard or soft index (following Donihue et al., 2015. See Appendix 2) to the taxa recovered from the stomach contents and the insect traps and calculated the same selection index for soft and hard prey taxa.

### 2.1 Statistical analyses

I used linear mixed effects models to test for differences in behavior, morphology, performance, and diet between wall and nonwall populations. The mixed effects models were evaluated using the LME command within the NLME (v3.1‐121; 2015) package in R (v3.1.2; 2014). Each model included the binary wall/nonwall as a fixed effect, site identity as a random effect, and for relative morphological and performance measures, SVL as a covariate (see Tables 1 through 3 for complete model specifications). The morphological, performance, and diet data were not normally distributed, so morphology and performance were Log_10_^−^transformed and diet proportion data were arcsine transformed before all analyses. Whenever SVL was added as a covariate, it was standardized to have a mean of zero so as to make the estimates of each response variable directly interpretable (standardized value = initial value – global mean value). For morphology and performance, I analyzed males and females independently to reduce interactions in the models. Finally, to assign *p*‐values appropriate for the unbalanced experimental design (Langsrud, 2003), I used type II ANOVAs (CAR package v2.0‐25) to calculate Wald chi‐square values for the wall fixed effect. All graphs were constructed in JMP (v11.2.0. SAS Institute Inc. 2013).

**Table 1:** Results of three models testing for differences in SVL (a), relative (i.e., standardized by including SVL as a covariate) head and limb morphometrics (b), and bite force (c) between lizards living on wall and nonwall sites. An (*) reflects significance at the p< 0.05 level

	Males				Females			
	*N*	*χ* ^2^	*df*	*p*	*N*	*χ* ^2^	*df*	*p*
Model (a): ~ Wall | Site								
SVL	175	9.0168	1	.002675*	149	4.3432	1	.03716*
Model (b): ~ Wall + SVL | Site								
Head Width	175	3.6094	1	.05745	149	12.671	1	.00037*
Head Length	175	0.4429	1	.5057	149	2.5928	1	.1074
Hindlimb: Forelimb Ratio	175	4.6627	1	.03082*	149	3.2719	1	.07048
Model (c): ~ Wall + SVL + Bite Plate Distance | Site								
Bite Force	166	13.795	1	.0002*	135	0.3091	1	.5782

## 3 Results

**Figure 2:**
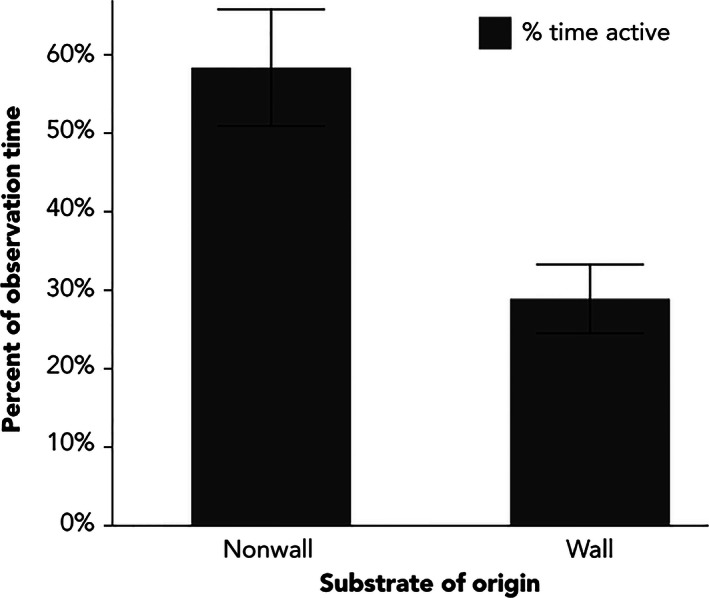
The average percent time that lizards from wall and nonwall sites spent active during observation. Standard error bars have been included

I found significant differences in behavior, morphology, performance, and diet among lizard populations inhabiting wall and nonwall habitats. As predicted, lizards on walls spent approximately 70% of their time sedentary, whereas lizards in nonwall sites were significantly more active ($\chi_{1,74}^{2}$ = 13.32, *p *=* *.0003), spending approximately 60% of their time in motion (Figure 2).

**Figure 3:**
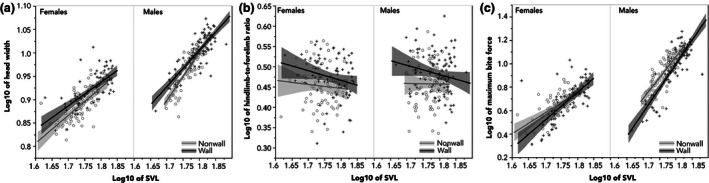
Variability in the relative size (standardized by SVL) of head width (a), the ratio of hindlimb to forelimb length (b), and the maximum bite capacity (c) among lizards from wall (dark lines, plus signs) and nonwall (light lines, circles) sites. In all instances, shaded regions reflect the 95% confidence interval around the best fit line

The SVL of both male and female lizards in nonwall sites was significantly smaller (hereafter all effect sizes related as mean ± *SE*. Wall males: 62.4 ± 0.6 mm, nonwall males: 58.1 ± 0.4 mm; wall females: 59.2 ± 0.7 mm, nonwall females: 55.0 ± 0.6 mm) than lizards at wall sites (Table 1). I hypothesized that the sit‐and‐wait lizards would have wider heads, most likely to facilitate ingestion of active prey. I found that, relative to their body size (i.e., with SVL as a covariate), female wall lizards had proportionally wider heads (wall females: 8.4 ± 0.08 mm, nonwall females: 7.8 ± 0.07 mm), males marginally so (wall males: 10.0 ± 0.09, nonwall males: 9.2 ± 0.09; Figure 3a, Table 1). I predicted longer heads among the active foragers, but did not detect differences in relative head length between the populations in either setting (Table 1). Finally, I predicted that active foraging lizards would have a lower hindlimb‐to‐forelimb ration. Indeed, I found that males had proportionally larger hindlimb‐to‐forelimb ratios at wall sites than did male lizards at nonwall sites (Figure 3b, Table 1). The same pattern was marginally significant for females (Figure 3b, Table 1).

I found strongly significant differences in male bite force between the two habitat types. Contrary to expectations for their foraging mode, males on rock walls bit significantly less hard than lizards from nonwall sites when accounting for differences in body size (Figure 3c, Table 1). No difference however was observed in females (Figure 3c, Table 1).

**Figure 4:**
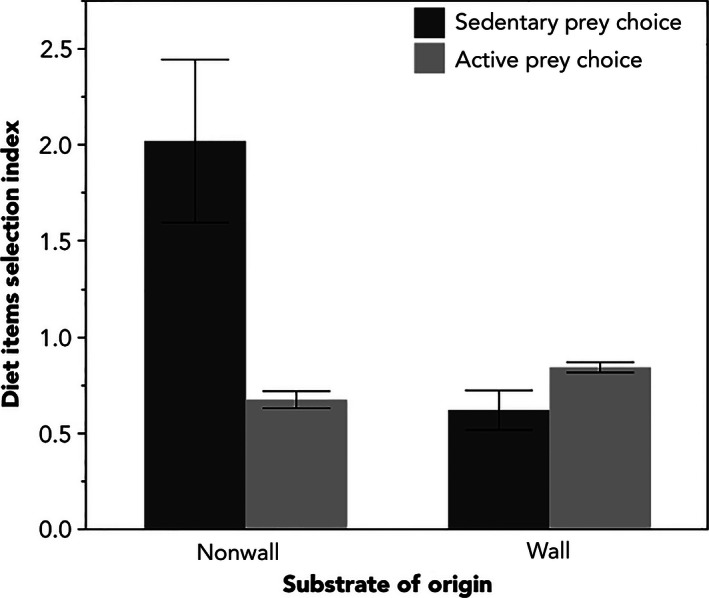
The average diet selection index of lizards from wall and nonwall sites for active prey taxa (light gray) and relatively sedentary prey taxa (dark gray). Standard error bars have been included

The available invertebrate prey at the two sites was very similar. I found no differences in the availability of fast, or sedentary insect taxa between the habitat types in either trap type—pitfall or sticky traps (Table 2). However, I found significant differences in the relative proportion of fast or slow insect taxa consumed by lizards in each habitat type. As expected for their foraging mode, lizards on walls tended to consume a significantly higher proportion of active taxa, whereas lizards in nonwall sites consumed a significantly higher proportion of sedentary taxa (Figure 4, Table 3). This pattern was strongly driven by male lizards (Table 3). A foraging selection index of one implies that animals are eating approximately in the proportion available to them in their surroundings (Loehle & Rittenhouse, 1982). I calculated 95% confidence intervals around the means of the selection index for active and sedentary taxa for both wall and nonwall lizards and found that only nonwall selection for sedentary taxa differed significantly from one.

**Table 2:** Results of LME models comparing the availability of fast, slow, hard, and soft prey taxa at wall and nonwall sites. An (*) reflects significance at the p< 0.05 level

Model	~Wall|Site			
	*N*	*χ* ^2^	*df*	*p*
Proportion of fast prey taxa available	134	0.5193	1	.4711
Proportion of slow prey taxa available	134	1.7273	1	.1888
Proportion of hard prey taxa available	134	2.7638	1	.0964
Proportion of soft prey taxa available	134	7.7128	1	.0055

**Table 3:** Results of LME models testing for differences in the proportion of fast or slow, hard, or soft prey taxa consumed by animals at wall and nonwall sites. Comparisons show differences between all animals, and males or females separately between wall and nonwall sites

Model	~ Wall|Site											
	All Animals				Males				Females			
	*N*	*χ* ^2^	*df*	*p*	*N*	*χ* ^2^	*df*	*p*	*N*	*χ* ^2^	*df*	*p*
Proportion of fast taxa consumed	134	8.7429	1	.003108*	90	8.8085	1	.00446*	44	3.5136	1	.06087
Proportion of slow taxa consumed	134	18	1	.00002*	90	13.895	1	.000193*	44	0.8809	1	.348
Proportion of hard taxa consumed	134	0.188	1	.6646	90	0.1383	1	.71	44	0.8632	1	.3528
Proportion of soft taxa consumed	134	0.1588	1	.6902	90	0.5248	1	.4688	44	0.016	1	.8994

Lastly, I found that while there was no difference in the number of hard insect taxa caught in traps at the wall and nonwall habitats, I caught more soft taxa in the nonwall insect traps (Table 2). Nonetheless, the lizards showed no preference for hard or soft taxa in either habitat type (Table 3).

## 4 Discussion

The lizard *P. erhardii* provides evidence for intraspecific foraging mode switching with accompanying shifts in many traits predicted by the foraging syndrome hypothesis. Lizards from wall sites were significantly larger than lizards from nonwall sites. Body proportions were also different: relative head width and hindlimb‐to‐forelimb ratios were generally greater for lizards on walls. These head shape differences however did not drive corresponding bite force differences; male lizards from nonwall sites bit significantly harder than males from wall sites. Finally, although the available insect community was very similar, the diet of lizards on walls contained a larger proportion of active prey taxa, whereas lizards from nonwall sites ate a higher proportion of sedentary prey. Many of these differences make sense in light of the significant behavioral shift between populations on walls, which remain relatively sedentary, in contrast to the lizards in nonwall sites that are substantially more active. This demonstrates that foraging behavior can switch as a result of human‐built habitat structure and variability in foraging mode‐associated traits can occur intraspecifically on a microgeographic scale.

### 4.1 Testing predictions of traits that vary with foraging mode

Analysis of percent of time in motion, a metric for differentiating between foraging modes (Perry, 2007), revealed that the percent of time lizards on rock walls spent in motion was on average 30%, making them relatively sit and wait in comparison with the nonwall lizards that spent approximately twice that amount of time active (Figure 2). These conclusions are based on behavior recordings that lasted two minutes on average, but ranged between 1 and 4 min. While longer recording times are preferred (Perry, 2007), the shorter recording times in this study stem from logistical constraints of recording continuous behavior of nonwall lizards that are very active and wide ranging, precluding observation without disruption. As such, only 26 video recordings among the nonwall sites met my 60‐s minimum continuous viewing threshold. The bias from this, however, favors relatively stationary lizards and leads to a conservative measure of activity level. As a consequence, it favors a greater likelihood of finding no difference between habitats than exacerbating the likelihood of finding a difference. Thus, I interpret the difference in foraging modes between habitats to be a real effect rather than an artifact of truncated video recording. Detecting stationary lizards in complex habitat is difficult and may have led to missed sedentary individuals at wall and nonwall sites. This risk was mitigated by carefully searching for all subjects, active and stationary, and by filming during the highest daily activity period for the species. I believe this potential bias did not significantly affect these results.

Lizard populations living in different habitat structures often display morphological differences facilitating performance in those habitats (Calsbeek & Irschick, 2007; Kohlsdorf & Navas, 2007; Vanhooydonck & Van Damme, 2003; Winchell, Reynolds, Prado‐Irwin, Puente‐Rolón, & Revell, 2016). For example, previous researchers have demonstrated in multiple lineages that lizards living in rocky habitats often exhibit morphological adaptations for navigating that habitat structure (Goodman, 2007; Kohlsdorf & Navas, 2007; Revell et al., 2007). Similarly, I found that lizards from wall and nonwall habitats had different head and limb proportions (Figure 3). The high hindlimb‐to‐forelimb ratio predicted for the sit‐and‐wait wall lizard populations has been demonstrated to be an adaptation for fast bursts of acceleration to capture prey (Huey & Pianka, 1981; Miles et al., 2007; Thompson & Withers, 1997). This is corroborated in a related study in which I found that lizards living on walls displayed a strong behavioral shift, increasing their jumping propensity (Donihue, 2016), which may be an adaptation for catching active prey in the wall habitat.

Head width is larger among many species of sit‐and‐wait predators (McBrayer & Corbin, 2007). This adaptation is largely attributed to decreasing prey‐handling time and increasing the lizards' capacity to consume larger prey items (McBrayer & Corbin, 2007; Vanhooydonck et al., 2007). This pattern was strongly apparent among female lizards (Figure 3) and trending among male lizards. I did not detect a difference in head length for either sex belonging to either population.

Other studies have shown that head size is closely correlated with maximum bite capacity in lizards (Donihue et al., 2015; Herrel et al., 1999; Huyghe, Herrel, Adriaens, Tadic, & Van Damme, 2009; Sagonas et al., 2014). Furthermore, bite force has been demonstrated to be larger among sit‐and‐wait predators, again to decrease prey‐handling time (Herrel et al., 2004; Vanhooydonck et al., 2007). Counter to the predictions, the maximum bite force of male lizards from wall habitats was weaker than that of males from nonwall sites when standardizing for the differences in SVL. One potential explanation is that while the insect taxa between sites may not have differed in activity levels, they may have differed in average hardness, which has been shown to influence lizard bite force elsewhere (Herrel & De Vree, 2009; Herrel et al., 2004, 2008). To explore this hypothesis, I re‐indexed the stomach contents and trap contents according to relative hardness indexes (following Donihue et al., 2015). While there was no difference in the availability of hard insect taxa between the wall and nonwall habitats, I caught more soft taxa in the nonwall insect traps. However, the wall and nonwall lizard populations, despite bite force differences, showed no preference for hard or soft taxa in either habitat type. This result is in contrast with other inter‐ and intraspecific lizard studies demonstrating a positive relationship between lizard bite force and the proportion of hard diet items (Des Roches, Brinkmeyer, Harmon, & Rosenblum, 2015; Verwaijen, Van Damme, & Herrel, 2002). These results suggest that the difference in bite force was not driven by differences in the available prey or differences in prey selection between the populations.

While this pattern in maximum bite force does not follow the predictions from previous interspecific foraging mode studies (Herrel et al., 2004; Vanhooydonck et al., 2007), it is consistent with a previous study showing *P. erhardii* bite force is less related to diet and more related to the local intraspecific competitive landscape (Donihue et al., 2015). While tests of this hypothesis remain to be conducted, other studies have documented significant intraspecific aggression in closely related active foraging *Podarcis* species (Lailvaux, Huyghe, & Van Damme, 2012; Pafilis, Meiri, Foufopoulos, & Valakos, 2009), which may explain the proportional increase in bite force among the active foraging male populations at nonwall sites.

I found stark differences in diet between lizards at wall and nonwall sites, as predicted from previous intraspecific studies (Huey & Pianka, 1981; Vanhooydonck et al., 2007). While lizards on walls selected a nearly equal proportion of active and sedentary insect taxa, the active foraging lizards from nonwall sites ate a much higher proportion of sedentary taxa (Figure 4). Because the 95% confidence intervals around the average proportion of sedentary taxa consumed by nonwall lizard populations was greater than one (Loehle & Rittenhouse, 1982), we can infer that these lizards were actively foraging for sedentary taxa in greater proportion than they were detected in traps and reflect a significant dietary shift between the wall and nonwall lizard populations. Pitfall and sticky insect traps undersample relatively sedentary taxa, and while this will bias the availability data, this bias is consistent between sites making these results still comparable.

These intraspecific differences in morphology, performance, and diet according to habitat context are commensurate with interspecific studies comparing foraging mode related traits. The activity level difference between wall lizards (30% time in motion) relative to nonwall lizards (60% time in motion) is comparable with foraging behavior variability at the genus and family level published in a review of lizard foraging behavior (Perry, 2007). The differences in hindlimb‐to‐forelimb ratio found between wall and nonwall populations are also consistent with interspecific comparisons between sit‐and‐wait and active foraging predators (Thompson & Withers, 1997). Finally, the difference in selection for sedentary or active taxa seen in the diets of the wall and nonwall lizards fall within the same range observed in a study contrasting the diet of 14 Lacertid lizards with varying foraging modes (Vanhooydonck et al., 2007). These results demonstrate that foraging mode syndromes can shift intraspecifically according to ecological context.

## 5 Conclusion

This study demonstrates that foraging mode is not typological for a species. Lacertids are considered a clade of active foraging species (Verwaijen & Van Damme, 2007), and indeed, the *P. erhardii* populations on Naxos from habitats that reflect the prehuman landscape in Greece (Grove & Rackham, 2003; Hughes, 2005) were active foragers. Those lizards however that had colonized the human‐built rock walls on Naxos have changed their foraging mode to be substantially more sit and wait. With this change in mode has come a fundamental shift in diet. Furthermore, this change has been accompanied by several companion traits, specifically head width among females, to lesser extent males, and hindlimb‐to‐forelimb ratio for both sexes—that should facilitate active prey capture and prey handling. These changes were predicted by the foraging syndrome hypothesis (McLaughlin, 1989) and demonstrate its utility, independent of confounding phylogenetic relatedness.

Whether this variability in foraging‐related traits is genetic or plastic remains unknown. While plasticity has been demonstrated in several lizard morphological and performance traits (Losos, 2009), the potential for microgeographic local adaptation to explain such variation is increasingly being recognized (Richardson et al., 2014). In this system, due to the close proximity of the habitat types, it is possible that lizards are actively selecting habitats where their fitness is maximized, resulting in local adaptation (Richardson et al., 2014). Additional genetic work will have to be performed to determine the relatedness and extent of gene flow between these populations.

Animal foraging mode is a functional trait (sensu Schmitz, Buchkowski, Burghardt, & Donihue, 2015) with the potential to have cascading effects on the dynamics of an ecosystem (Bolnick et al., 2011; Schmitz, 2008). This study demonstrates that intraspecific foraging mode variability can result in differential invertebrate predation between adjacent habitats on the Greek Island of Naxos and opens the question for future study of whether those predation differences cascade to affect plants and broader ecological dynamics on these islands. Furthermore, this study demonstrates the effects that human‐built infrastructure can have on the dynamics of an ecosystem (Donihue & Lambert, 2014). With the increasing prevalence of human land use and the increasing examples of microgeographic local adaptation, we should expect changes in functional traits and cascading trait‐mediated effects in human‐dominated ecosystems.

## Conflict of Interest

I have no conflict of interest to declare.

## Appendix 1 The coordinates of the 10 study sites on Naxos including their site name and whether they had or did not have a rock wall. All coordinates are from Google Earth (2015) using WGS84 datum

### 1.1

**Table:**

Site	Wall	Latitude	Longitude
Alyko 1	No Wall	36 59.074′ N	25 23.420′ E
Alyko 2	No Wall	36 58.053′ N	25 23.026′ E
Alyko 3	No Wall	36 58.058′ N	25 23.026′ E
Mikri Vigla	No Wall	37 01.489′ N	25 23.184′ E
Rachi	No Wall	37 00.883′ N	25 24.179′ E
Demarionas	Wall	37 03.230′ N	25 28.383′ E
East Moni	Wall	37 06.328′ N	25 22.644′ E
Sagri	Wall	37 03.201′ N	25 25.695′ E
South Moni	Wall	37 05.076′ N	25 29.688′ E
West Moni	Wall	37 05.074′ N	25 29.688′ E

## Appendix 2 Invertebrates found in lizard stomach contents along with their assigned hardness index and activity level index

### 2.1

**Table:**

	Hardness Index	Activity Index
Hemiptera	Soft	Fast
Lepidoptera	Soft	Fast
Araneae	Soft	Fast
Lepidoptera Larvae	Soft	Slow
Coleoptera larvae	Soft	Slow
Coleoptera	Hard	Fast
Hymenoptera	Hard	Fast
Orthoptera	Hard	Fast
Acari	Hard	Slow
Gastropoda	Hard	Slow
